# GR3027 reversal of neurosteroid-induced, GABA-A receptor-mediated inhibition of human brain function: an allopregnanolone challenge study

**DOI:** 10.1007/s00213-018-4864-1

**Published:** 2018-02-28

**Authors:** Maja Johansson, Maria Månsson, Lars-Eric Lins, Bruce Scharschmidt, Magnus Doverskog, Torbjörn Bäckström

**Affiliations:** 1 Umecrine Cognition AB, Karolinska Institutet Science Park, Fogdevreten 2, SE-171 65 Solna, Sweden; 2 Department of Clinical Sciences, Obstetrics and Gynecology, Umeå University, SE-901 87 Umeå, Sweden

**Keywords:** GR3027, Allopregnanolone, Clinical trial, Saccadic eye movement, Sedation

## Abstract

**Rationale:**

GR3027 is a novel small molecule GABA-A receptor-modulating steroid antagonist, which in non-clinical studies has shown promise for treatment of human disorders due to allosteric over-activation of GABA-A receptors by neurosteroids, such as allopregnanolone. We here studied its safety, pharmacokinetics, and ability to inhibit allopregnanolone effects in humans.

**Methods:**

Safety and pharmacokinetics were studied in healthy adult males receiving ascending single or multiple oral GR3027 vs. placebo. GR3027-mediated reversal of allopregnanolone effect on maximal saccadic eye velocity (SEV), and self-rated somnolence was studied in a double-blind, placebo-controlled, three-part cross-over study in which 3 or 30 mg oral GR3027 preceded 0.05 mg/kg of i.v. allopregnanolone.

**Results:**

GR3027 was well tolerated, adverse events were generally mild and transient, and no dose-limiting toxicity or grade 3 adverse events were observed up to the highest single (200 mg) or multiple (100 mg every 12 h for 5 days) doses. The maximum concentration (*C*_max_) and systemic exposure (area under the plasma concentration-time curve from dose extrapolated to infinity [AUC_0–∞_] and/or AUC during the dosing interval [AUC_τ_]) varied linearly with dose; with dose-dependent accumulation ratios of 1.3–1.6. Allopregnanolone decreased SEV and induced somnolence in most, but not all subjects. By predefined analyses, 30 mg GR3027 significantly inhibited allopregnanolone-induced decrease in SEV (*p* = 0.03); 3 and 30 mg GR3027 non-significantly inhibited allopregnanolone-induced sedation. By post hoc analyses restricted to subjects with allopregnanolone-induced changes and the time period over which they occurred, GR3027 dose dependently inhibited allopregnanolone-induced decrease in SEV (*p* = 0.04 at 30 mg, non-significant at 3 mg) and allopregnanolone-induced sedation (*p* = 0.01/0.05 at 3/30 mg doses).

**Conclusion:**

Oral GR3027 mitigates inhibition of brain function induced by allopregnanolone at doses which are clinically well tolerated and associated with linear pharmacokinetics.

**Electronic supplementary material:**

The online version of this article (10.1007/s00213-018-4864-1) contains supplementary material, which is available to authorized users.

## Introduction

The GABA system is the major inhibitory neurotransmitter system in the brain. It regulates diverse CNS functions including learning, memory, sedation, and sleep and is a validated pharmacological target (Olsen and Sieghart 2008). While a variety of GABA-A agonist drugs have been approved, attempts to develop GABA-A antagonists have been hindered by their propensity to induce seizures. Indeed, pentylenetetrazol, a GABA-A antagonist, is commonly used as an experimental CNS convulsant (Adeoluwa et al. 2016).

GABA-A receptors are ionotropic heteropentameric assemblies derived from a family of 19 members (6α, 3β, 3γ, δ, ε, θ, π, 3ρ), with 20 distinct configurations described (Olsen and Sieghart 2008). Their functional characteristics vary depending on receptor subtype and localization within the brain and within the neuron. For example, extrasynaptic α5 subunit-containing GABA-A receptors have a well-established role in the hippocampus, a brain area involved in learning and memory, and represent an attractive target for memory-enhancing drugs with antagonizing properties (Mohler and Rudolph 2017). By contrast, activation of α1 subunit-containing GABA-A receptors, which are more dispersed and most often located within the synapse, induces sedation and sleep (Mohler 2002). The GABA system is also involved in sleep regulation (Saper et al. 2010; Scammell et al. 2017). Most sedative/hypnotics used for insomnia target the GABA-A receptor (Winsky-Sommerer 2009), and activation of GABA-A receptors in the posterior hypothalamus promotes sleep and contributes to both homeostatic and circadian sleep regulation (Volgin et al. 2014).

In addition to GABA, endogenous 3α,5α- and 3α,5β-reduced metabolites of progesterone such as allopregnanolone (3α-hydroxy-5α-pregnane-20-one) and THDOC (3α,5α-tetrahydrodeoxycorticosterone), commonly referred to as neurosteroids, act as strong positive allosteric modulators of the GABA-mediated activation of both synaptic and extrasynaptic GABA-A receptors (Majewska et al. 1986), thus enhancing both phasic and tonic inhibition. Allopregnanolone is a potent anesthetic and induces an inhibitory GABAergic tone in the CNS that alters the sleep/wake cycle and impairs memory and learning. Inhibition of the effects of neurosteroids on the GABA-A receptor holds promise as a therapeutic approach to revere GABAergic-mediated inhibition of CNS function without the analeptic effects associated with GABA-A receptor antagonists (Johansson et al. 2016). GR3027 is a novel investigational drug that has been shown *in vitro* to selectively antagonize the positive modulation of α1- and α5-subunit-containing GABA-A receptors by neurosteroids, i.e., potentially influencing both phasic and tonic inhibition, without antagonizing the effects of GABA and in vivo to restore spatial learning and motor coordination in two animal models of hepatic encephalopathy (Johansson et al. 2015; Johansson et al. 2016). In the present studies, we have examined its safety, tolerability, and pharmacokinetics in humans during single and multiple ascending dose (SAD and MAD) studies in healthy adults as well as its ability to enter the CNS and exhibit GABA-A receptor-modulating steroid antagonist properties in the context of an allopregnanolone challenge using maximal saccadic eye velocity (SEV) and sedation/somnolence as pharmacodynamic markers.

Saccadic eye velocity is used to assess the effects of GABA-A receptor agonists such as allopregnanolone or benzodiazepines (Ball et al. 1991; Timby et al. 2006). The saccade is the fast movement of the eye when the gaze is shifted to a new peripheral visual stimulus. When a new visual target is presented, the gaze, after a latency period, moves towards the new target (Carpenter 2000). The maximal speed reached during the saccade, i.e., the SEV, is dependent on the angle between the targets, is regulated by the activity of the GABA-A receptor, and is not under conscious control (Ball et al. 1991). SEV is, therefore, a sensitive and quantifiable measure of GABA-A receptor modulation by compounds like allopregnanolone or benzodiazepines (Ball et al. 1991; Carpenter 2000; Moller et al. 2016; Timby et al. 2006). A GABA-A-mediated decrease in SEV, for example by allopregnanolone, is generally accompanied by increased sedation/somnolence (Timby et al. 2006; van Broekhoven et al. 2007).

The present studies demonstrate that GR3027, a novel oral GABA-A receptor-modulating steroid antagonist, enters the brain and inhibits the effect of the neurosteroid allopregnanolone in humans at doses which are well within the range that exhibits excellent tolerability and pharmacokinetics. The studies build on prior work exploring the ability of isoallopregnanolone (an endogenous neurosteroid antagonist) to antagonize the effects of allopregnanolone on SEV and sedation in animals and humans (Backstrom et al. 2005; Bengtsson et al. 2015).

## Methods

### Study design

The first in human GR3027 SAD study (part 1 of EudraCT 2015-004911-19) and the 5-day MAD study (EudraCT 2016-003651-30) were prospective, randomized, double-blind and placebo-controlled and designed to assess the safety, tolerability, and pharmacokinetic characteristics of single and multiple ascending oral doses of GR3027 in healthy male volunteers. GR3027 (SAD 1, 3, 10, 30, 100, and 200 mg; MAD 50 mg/day, 50 or 100 mg twice daily (BID)) was administered orally in the morning or every 12 h.

The antagonist study (part 2 of EudraCT 2015-004911-19) was randomized and placebo-controlled and had a double-blind three-part cross-over design, with at least 1 week between test occasions. In the morning, study subjects orally received placebo (A), 3 mg GR3027 (B), or 30 mg GR3027 (C) followed 90 min later by an intravenous (i.v.) allopregnanolone challenge and underwent repeated measurement of SEV and sedation/somnolence before administrations and for 3 h thereafter. Subjects were randomized concerning the order of the oral treatments, so that three subjects followed the same sequence, i.e., ABC, ACB, BAC, BCA, CAB, and CBA, respectively.

The studies were performed in accordance with ethical principles that have their origin in the Declaration of Helsinki and are consistent with ICH/Good Clinical Practice (GCP) E6(R1), European Union (EU) Clinical Trials Directive, and applicable local regulatory requirements. The study was approved by the Independent Ethics Committee in Uppsala, Sweden, and by the Swedish Medical Product Agency, and was performed at Clinical Trial Consultants AB (CTC) in Uppsala, Sweden.

### Subjects

Ninety (90) eligible healthy consenting male subjects (age 18–50 years) were included in the studies and randomized to treatment (GR3027 or placebo 6:2 in SAD and MAD protocols) or treatment sequence in the antagonist study (6 groups of 3 individuals). Attending subjects were recruited from the CTC database of persons interested in participating in clinical trials or by advertisements. All randomized subjects completed the SAD and MAD studies, while 17 subjects completed all study visits in the antagonist study and one subject was withdrawn when presenting at the clinic for the second treatment period. Withdrawal was due to moderate sadness (depressed mode), sleepiness (somnolence), and mild vertigo that were assessed as not related to study treatment.

Exclusion criteria included the following: present (assessed by medical examination, ECG, and laboratory values at screening) or history of any clinically significant disease or disorder, including anxiety (GAD-7 questionnaire); depression (PHQ-9 questionnaire and the The Columbia-Suicide Severity Rating Scale), which could either put the subject at risk or influence the results; use of any prescribed or non-prescribed medication including antacids, analgesics, herbal remedies, vitamins, and minerals within 2 weeks prior to study visits, except the occasional intake of paracetamol and nasal decongestants without cortisone or antihistamine for a maximum of 10 days; a history of alcohol abuse; positive screen for drugs of abuse or alcohol; and present or historic use of anabolic steroids. During the study, consumption of alcohol, coffee (SAD and antagonist study), xanthine- or taurine-containing products/beverages, and grapefruit or grapefruit-containing products was not allowed, nor was smoking or use of nicotine-containing products.

### Trial compounds

GR3027 (Umecrine Cognition AB, Sweden) was administered orally in gelatin capsules, 1 or 10 mg per capsule (RISE, Bioscience and Materials - Surface, Process and Formulation, Södertälje, Sweden). Placebo capsules contained excipients only and were of identical appearance as the GR3027 capsules.

The allopregnanolone (Umecrine AB, Sweden) formulation was made by Cobra Biologics Matfors, Sweden, in a concentration of 0.13 mg/mL allopregnanolone dissolved in an albumin solution (200 mg/mL).

### Experimental protocols

The screening visit included a physical examination, historical and baseline medical characteristics, and inclusion/exclusion criteria as well as signing of informed consent. Subjects were confined to the clinic from the evening before dosing until 24 h (SAD and antagonist study) or 48 h (MAD) post last dose. A follow-up visit was performed 5–10 days, or 21 days (MAD), after the last dose of GR3027.

Study days with PK sampling or SEV testing were preceded by an 8-h overnight fasting period before administration of the oral placebo or GR3027; food intake was at the earliest 2 h after dose. In the antagonist study, allopregnanolone (0.05 mg/kg body weight) was administered as an i.v. infusion (2 mg/min over ~ 1–2 min) 90 min after placebo/GR3027.

Vital signs, including body temperature, blood pressure, heart rate, and clinical chemistry (e.g., alkaline phosphatase, alanine aminotransferase, aspartate aminotransferase, bilirubin, creatinine), were followed during the experimental period.

#### Saccadic eye measurements

For calculation of SEV, saccadic eye measurements (SEM) were performed at pre-oral GR3027 dosing, pre-allopregnanolone dosing (− 10 min), and 10, 20, 40, 60, 80, 100, 120, 140, 160, and 180 min after allopregnanolone.

A *Saccadometer Plus* device (Ober Consulting Sp. z o.o, Poland) using direct infrared photo-oculography was used with visual stimuli 20 degrees apart horizontally. Measurements for an individual subject were performed at approximately the same time at each of the three study visits. At each time point, data were collected from 30 saccades. Not all saccades were evaluable. The saccade data used for analysis fulfilled the following prespecified criteria: were not rejected by the automatic validation, amplitude between 15 and 25 degrees, duration below 150 ms, and latency above 50 ms. Data from time points with less than 15 evaluable saccades were regarded as missing. For data analysis, the median SEV at each time point was used. The change in SEV induced by the allopregnanolone injection (ΔSEV) was calculated with the SEV measure 10 min before allopregnanolone as baseline value. ΔSEV was used as SEV varies greatly between individuals but has a high intra-individual stability, both within testing sessions and between testing days (Mercer et al. 1990; Wilson et al. 1993). For calculation of ΔSEV area under the curve (AUC_t_ ΔSEV), missing data points were imputed with the use of present data points just before and just after the missing point.

The response to allopregnanolone alone was evaluated at the study visit when oral placebo was given before the allopregnanolone injection. Allopregnanolone-responding subjects were defined as those showing a decrease in SEV during at least two succeeding measurements.

#### Sedation

Before each SEM time point, sedation was assessed by the subject using a visual analogue scale (VAS) with the lower end representing “Absence of sleepiness” (0 mm) and the upper end indicating “Falling asleep” (100 mm) and the value scored recorded. Data were treated similarly to SEV data, as described above; there were no missing data.

The response to allopregnanolone alone was evaluated at the study visit when it was preceded by oral placebo. Allopregnanolone-responding subjects were defined as those scoring an increase in sedation lasting for at least two measures in a row, with an increase of at least 10 mm in the peak.

#### Blood sampling

Samples were collected pre-dose and at 20, 40, 60, and 90 min and 2, 3, 4, 6, 9, 12, 18, and 24 h following dosing in the SAD protocol and the MAD 50 mg/day group and after last dose in all MAD groups. With BID dosing, the 18- and 24-h time points were omitted after the first dose. In the SAD study, 48-h samples were obtained after the higher single doses and in the MAD study after the last dose. In the MAD study, pre-dose samples were collected all days.

In the antagonist study, blood samples for analysis of serum levels of allopregnanolone (− 10, 10, 20, 40, 60, 120, and 180 min) and plasma levels of GR3027 (− 10, 60, 120, 180 min) were collected through an indwelling venous catheter.

Collected samples were stored at minus 70 °C and shipped on dry ice for analyses.

### Analyses of GR3027 and allopregnanolone concentrations

The plasma concentration of GR3027 and serum concentration of allopregnanolone were quantified by UPLC/MS/MS Waters Acquity, C18 columns, and Waters XEVO-TQ-S triple quadrupole mass spectrometers at the National Veterinary Institute (SVA; Uppsala, Sweden; GR3027) and at Admescope Oy (Oulu, Finland, allopregnanolone). As internal standards, D4-GR3027 and D5-allopregnanolone were used, respectively. For analysis of allopregnanolone, oxime derivatization was used to increase sensitivity (Keski-Rahkonen et al. 2011). Standard and QC samples were prepared into human blank plasma/serum. The detection limit for GR3027 was 1 ng/mL and for allopregnanolone 0.05 ng/mL (0.15 nmol/L).

### Pharmacokinetic analyses

The following pharmacokinetic parameters were evaluated using non-compartmental analysis and the software Phoenix WinNonlin® version 6.3 or later (Pharsight Corporation, USA): maximal concentration (*C*_max_), time point for *C*_max_ (*T*_max_), terminal half-life (*T*_1/2_), area under the curve extrapolated to infinity (AUC_0–>∞_), area under the curve during dosing interval (AUC_τ_), and total apparent body clearance following extravascular administration (CL/F). Dose proportionality was calculated for the SAD study based on AUC_0–>∞_ and *C*_max_, and in the MAD study on steady-state AUC_τ_. In the MAD study, the accumulation ratio (*R*_A,AUC_) between the first and last doses was calculated as (steady-state AUC_τ_ ∕ first-dose AUC_τ_).

### Statistical analyses

Statistical calculations were with SAS® (Version 9.4, SAS Institute Inc., Cary, NC, USA) and IBM SPSS statistics (Version 24, IBM).

Treatment differences based on AUC_t_ ΔSEV and AUC_t_ ΔVAS (prespecified, assuming the duration of the allopregnanolone effect to be 180 min), the same parameters divided by the actual allopregnanolone AUC in each subject (prespecified), allopregnanolone exposures (AUC allopregnanolone) with different oral pretreatment (prespecified), and SEV and VAS measures before allopregnanolone were analyzed using Wilcoxon’s signed-rank test. For non-related samples, i.e., allopregnanolone concentration vs. body mass index (BMI), the Mann-Whitney *U* test was used. Estimates of the treatment effect were also performed with a prespecified mixed model repeated measure (MMRM) analysis with ΔSEV or ΔVAS as dependent variables and treatment, visit, and time point as fixed effect independent variables. The sample size of 18 for the allopregnanolone challenge study was based on power calculations indicated that 14 and 17 evaluable subjects were needed for 80 and 90% power, respectively, to detect a true difference in a two-sided test at the 5% significance level.

During statistical analysis of allopregnanolone-responding subjects, one subject showing an atypical response to allopregnanolone was excluded, i.e., for this subject, SEV continuously decreased and sedation continuously increased during the study visit.

## Results

### Subject recruitment and disposition

Of the 48 and 24 subjects who met all entry criteria and were enrolled in the SAD and MAD protocols, respectively, all completed the study and were included in the final analyses. Among the 18 subjects enrolled to the antagonist study, 1 subject was excluded because of an adverse event (AE) judged unrelated to treatment, while 17 completed the protocol and were included in the prespecified analyses (Supplementary Fig. 1). Post hoc analyses which excluded patients who exhibited either no effect or an atypical effect of allopregnanolone included 11 of the 17 subjects for SEV (65%) and 8 of 17 subjects for sedation (47%).

### Safety and tolerability

Single- and multiple-dose oral administration of GR3027 was generally well tolerated by the subjects, no serious adverse events (SAEs) occurred, and all AEs were transient and classified as mild (grade 1) or moderate (grade 2). No clinically significant abnormal physical examination findings or clinical chemistry values were reported at any of the time points assessed.

#### SAD

Treatment-emergent AEs possibly related to treatment were reported by four subjects (11%) receiving GR3027, while no possibly or probably related AEs were reported by subjects that received placebo (0%). No increased frequency or intensity of events was seen by increasing GR3027 dose. The possibly related AEs reported were headache (*n* = 3), pruritus (*n* = 1), and rash (*n* = 1).

#### MAD

Thirteen treatment-emergent AEs possibly or probably related to treatment were reported by eight subjects receiving GR3027 (44%): three subjects (50%) treated with 50 mg GR3027 once daily, one subject (17%) treated with 50 mg GR3027 twice daily (BID), and four subjects (67%) treated with 100 mg GR3027 BID. Five treatment-emergent AEs possibly or probably related to treatment were reported by two subjects (33.3%) that received placebo. Most of the symptoms were related to the gastrointestinal tract (50 mg, 2 AEs (2 subjects); 50 mg BID, 0 AE; 100 mg BID, 5 AEs (3 subjects); placebo, 2 AEs (1 subject)), all were of short duration (minutes to few hours) and most were graded as mild, with one moderate. There were also reports of fatigue (50 mg; *n* = 1), thirst (50 mg BID; *n* = 1), dizziness (100 mg BID; 1 mention each by 2 subjects), and headache (100 mg BID; 1 mention each by 2 subjects).

#### Agonist study

Four subjects experienced treatmen-emergent AEs possibly or probably related to treatment, i.e., allopregnanolone or GR3027 (24%). While receiving GR3027 plus allopregnanolone, headache (*n* = 1) and abdominal pain (*n* = 1) were reported, both during treatment with 3 mg GR3027. When treatment was with placebo plus allopregnanolone, headache was reported by three subjects.

### Pharmacokinetics

**Fig. 1:**
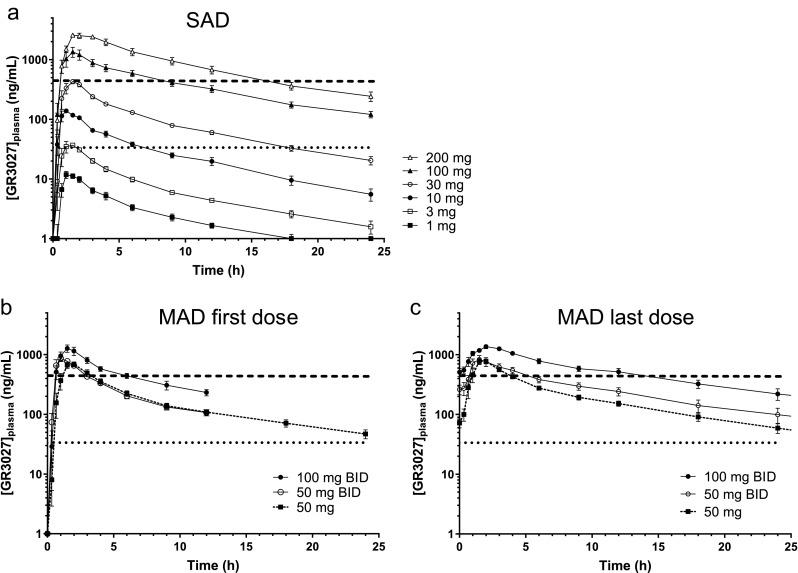
GR3027 plasma concentrations in the SAD and MAD studies. The three panels depict GR3027 concentrations in healthy adult males during SAD (**a**) and during days 1 (**b**) and 5 (**c**) of MAD, *n* = 6 for each dose. Horizontal lines represent the peak concentrations of GR3027 following the 3 and 30 mg doses used in the 3 h allopregnanolone challenge study. Values below 1 ng/mL are shown as 1 ng/mL (mean ± SEM)

After single dosing with GR3027, the plasma concentration increased proportionally to the increase in dose with proportionality constants *β* of 1.01 for *C*_max_ and of 1.12 for AUC_0–∞_ (Fig. 1a and supplementary Table 1). Dosing for 5 days twice daily resulted in a proportional increase in steady-state plasma GR3027 with 1.9 times increase in AUC_τ_ when the dose was doubled from 50 to 100 mg BID, and with mean (± SD) accumulation ratios of 1.3 ± 0.1 (50 mg once daily), 1.5 ± 0.3 (50 mg BID), and 1.6 ± 0.4 (100 mg BID) (Fig. 1b, c and supplementary Table 1).

### GR3027 antagonism of allopregnanolone-induced decreased SEV: prespecified analyses

**Fig. 2:**
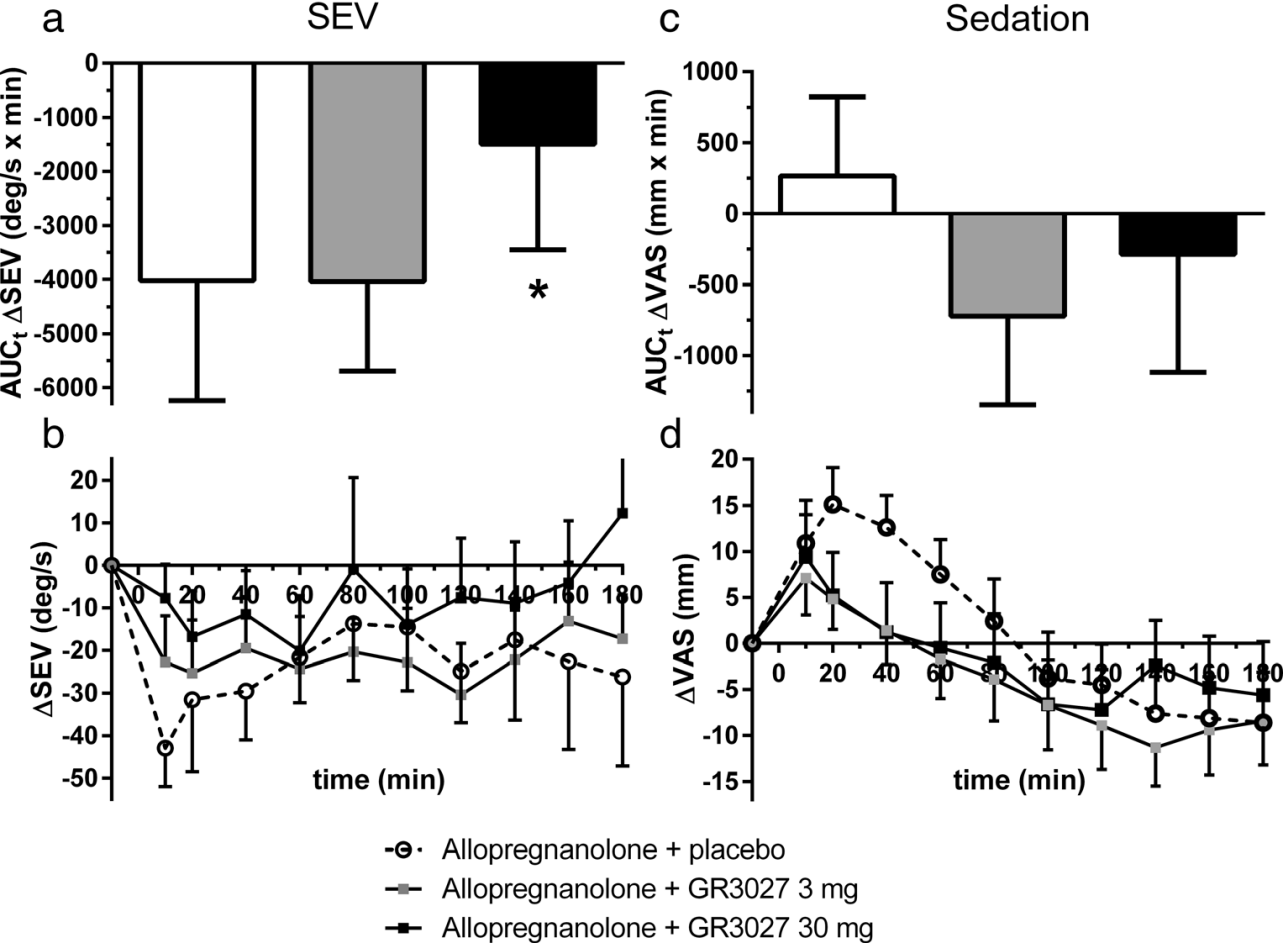
Effect of oral GR3027 on the allopregnanolone-induced change in maximal saccadic eye velocity and sedation, prespecified analyses with Wilcoxon’s signed-rank test including all evaluable data (*n* = 17 subjects) for the whole study period, up to 180 min after the allopregnanolone injection. (**a**) Area under curve for ΔSEV. (**b**) ΔSEV over time (mean ± SEM). (**c**) Area under curve for sedation. (**d**) Change in sedation over time (mean ± SEM). Note that 30 mg GR3027 significantly antagonized the allopregnanolone-induced change in SEV (*p* = 0.030), whereas 3 mg GR3027 did not (*p* = 0.89)

The allopregnanolone-induced change in SEV (placebo pretreatment; ΔSEV) analyzed as area under the curve (AUC) was − 4025 ± 2215 (min × deg/s); a similar AUC_t_ ΔSEV of − 4042 ± 1654 (min × deg/s) was found for 3 mg GR3027 pretreatment, while AUC_t_ ΔSEV for pretreatment with 30 mg GR3027 was − 1494 ± 1840 (min × deg/s). Thus, 30 mg GR3027 significantly antagonized the allopregnanolone-induced change in SEV (*p* = 0.030, Wilcoxon’s signed-rank test, Fig. 2(a)). There was no antagonism by the 3 mg GR3027 dose (*p* = 0.89).

The statistical significance for 30 mg GR3027 was also present in relation to the actual allopregnanolone exposure (AUC_t_ ΔSEV/AUC_t_ allopregnanolone). For allopregnanolone − 2.61 ± 1.51 (min ×deg/s/min × ng/mL) and − 0.94 ± 1.23 (min × deg/s/min × ng/mL) with 30 mg GR3027 (*p* = 0.041).

Allopregnanolone decreased SEV with a maximal effect of − 42.9 ± 9.1 deg/s at the first studied time point 10 min after the administration (Fig. 2(b), placebo treatment). The SEV reduction gradually diminished until 80 min after allopregnanolone but did not return to baseline. When study subjects received GR3027 before the allopregnanolone administration, there was also a decrease in SEV at the early time points with maximal decrease 20 min after the allopregnanolone administration, i.e., − 25.3 ± 12.6 deg/s (3 mg) and − 16.7 ± 10.2 deg/s (30 mg). With 30 mg GR3027, SEV was back to baseline at the end of the study period.

Statistical analyses over the whole study period (up to 180 min after allopregnanolone) with mixed model repeated measures of ΔSEV was inconclusive due to considerable variability that was not explained by the hypothesized model.

### Post hoc analyses of GR3027 antagonism of change in SEV among allopregnanolone-responding subjects during the responsive period

**Fig. 3:**
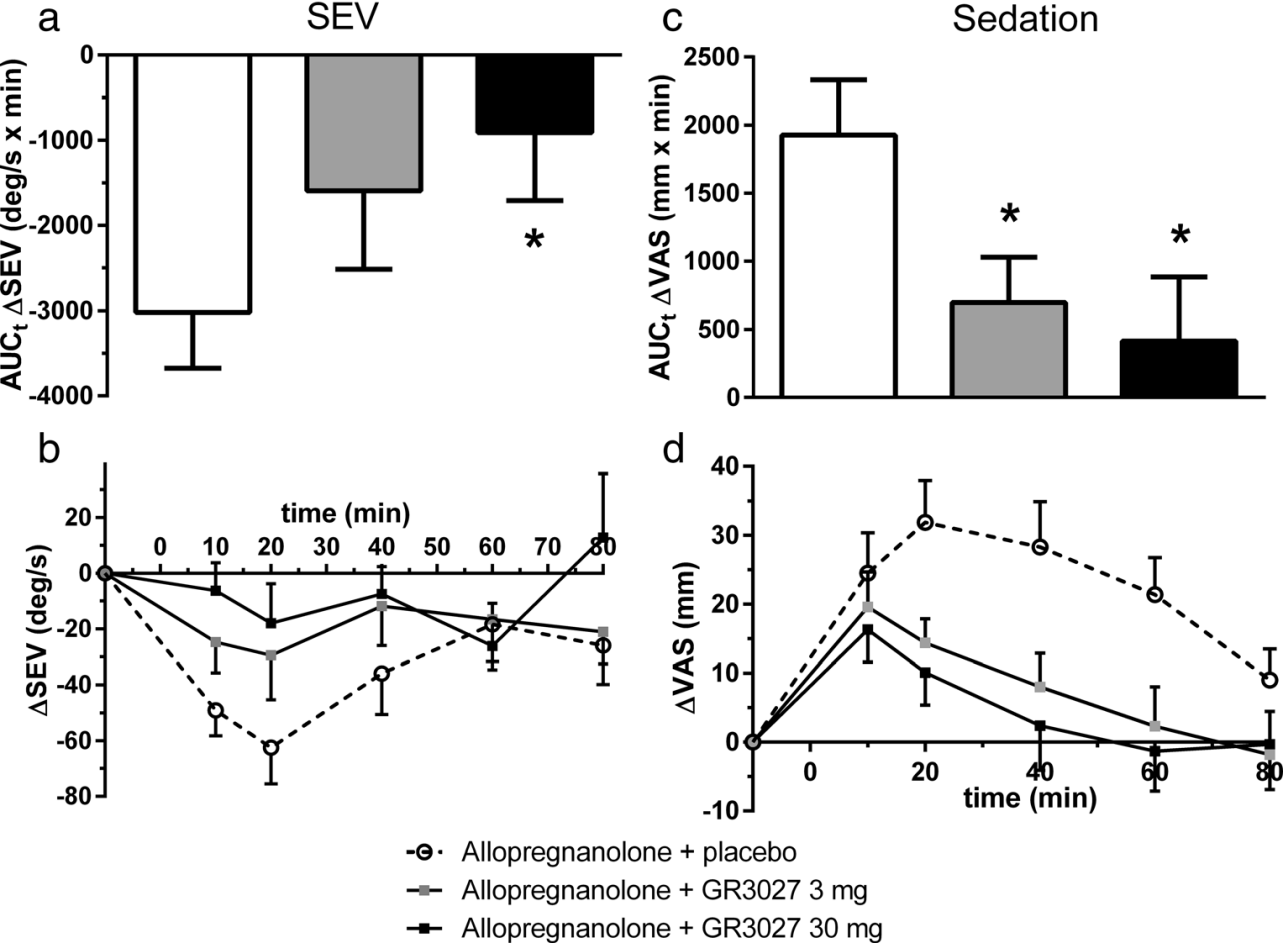
Effect of oral GR3027 on the allopregnanolone-induced change in maximal saccadic eye velocity and sedation in allopregnanolone-responding subjects (*n* = 11 for SEV and *n* = 8 for sedation), post hoc analyses during the responsive period, up to 80 min after the allopregnanolone injection. (**a**) Area under curve for ΔSEV. (**b**) ΔSEV over time (mean ± SEM). 30 mg GR3027 significantly inhibited the allopregnanolone-induced decrease in SEV (*p* = 0.04), whereas the diminution of the allopregnanolone effect by 3 mg GR3027 was non-significant (*p* = 0.29). (**c)** Area under curve for sedation. (**d)** Change in sedation over time (mean ± SEM). Both 3 (*p* = 0.012) and 30 mg (*p* = 0.05) GR3027 significantly inhibited the sedative effect

When subjects responding to the allopregnanolone injection with a decrease in SEV were studied separately, it was obvious that the SEV decrease induced by allopregnanolone lasted only approximately 80 min after the injection (Fig. 3(b)) rather than the 180 min assumed in the prespecified analyses. Statistical analysis of AUC_t_ ΔSEV from the responsive period (Fig. 3(a)) showed that 30 mg GR3027 significantly inhibited the allopregnanolone-induced decrease in SEV (*p* = 0.04), while the diminution of the allopregnanolone effect by 3 mg GR3027 was non-significant (*p* = 0.29). AUC_t_ ΔSEV for allopregnanolone was − 3018 ± 656 min × deg/s, while AUC_t_ ΔSEV with 3 mg GR3027 was − 1592 ± 924 min × deg/s and with 30 mg GR3027–909 ± 798 min × deg/s.

In this study population, allopregnanolone maximally decreased SEV by − 62.5 ± 13.0 deg/s at 20 min after allopregnanolone injection (Fig. 3b). GR3027 antagonized the allopregnanolone-induced changes so that the SEV measures at the same time point were − 29.5 ± 15.9 deg/s (3 mg GR3027) and − 18.0 ± 14.2 deg/s (30 mg GR3027).

### Absence of effect of GR3027 alone on SEV

On the three study occasions, SEV measured just prior to the oral capsules was 519 ± 19.6 deg/s (placebo), 520 ± 21.9 deg/s (3 mg GR3027), and 519 ± 25.0 deg/s (30 mg GR3027), and 90 min after the oral capsules but just prior to allopregnanolone was 532 ± 19.8 deg/s (placebo), 519 ± 15.7 deg/s (3 mg GR3027), and 508 ± 20.4 deg/s (30 mg GR3027). All measurements were similar and there was no evidence of change in SEV by GR3027 per se.

### GR3027 antagonism of allopregnanolone-induced sedation: prespecified analyses

There were no statistically significant changes in sedation by GR3027 when all subjects were included in the analysis of the whole study period (Fig. 2(c and d)).

Allopregnanolone increased the self-rated sedation (Fig. 2(d), placebo treatment), with the highest sedative score shown at the period 20 to 40 min after the injection of + 15.1 ± 5.5 and + 12.6 ± 5.0 mm, at 20 and 40 min respectively. Also, the sedative effect lasted for 80 min while thereafter the study subjects experienced even less sedation than before allopregnanolone. GR3027 (3 and 30 mg) appeared to decrease sedation induced by allopregnanolone; however, this effect was non-significant by the predefined statistical analyses. With the mixed model repeated measure analysis of change in sedation over time, convergence criteria for the hypothesized model were met but there was limited variability in the data (3 mg *p* = 0.67, 30 mg *p* = 0.90). No post hoc analysis was performed to explore additional interactions or parameters that could explain changes in sedation. Analysis of the area under the curve for change in sedation (AUC_t_ ΔVAS, Fig. 3a) or AUC_t_ ΔVAS divided by AUC_t_ allopregnanolone concentration showed neither any significant inhibition by GR3027 (3 mg, *p* = 0.31 and 0.28, respectively; 30 mg, *p* = 0.61 and 0.55, respectively).

### Post hoc analyses of GR3027 antagonism of sedation among allopregnanolone-responding subjects during the responsive period

For subjects responding to the allopregnanolone injection with sedation, the statistical analysis showed that both 3 and 30 mg GR3027 significantly inhibited the sedative effect (*p* = 0.012 and *p* = 0.05, respectively). The mean AUCs of sedation (AUC_t_ ΔVAS) after the different treatments were 1928 ± 404 min × mm (placebo), 698 ± 332 min × min (3 mg GR3027), and 416 ± 469 min × min (30 mg GR3027) (Fig. 3(c)).

After allopregnanolone, this study population scored a maximal increase of 31.9 ± 6.5 mm on the VAS scale for measure of sedation, with an increase above 20 mm for the period 10 to 60 min after allopregnanolone (Fig. 3(d)). With GR3027 pretreatment, the largest sedative increases were 19.6 ± 5.4 and 16.3 ± 5.0 mm, with 3 and 30 mg GR3027 respectively, and sedation thereafter quickly diminished.

### GR3027 by itself did not affect sedation

On the three study occasions, sedation levels were assessed just before oral GR3027: 24 ± 4.6 mm (placebo), 30 ± 4.3 mm (3 mg GR3027), and 26 ± 4.2 mm (30 mg GR3027), as well as 90 min after the oral treatment but prior to allopregnanolone: 27 ± 4.8 mm (placebo, *p* = 0.033), 30 ± 4.7 mm (3 mg GR3027, *p* = 0.11), and 32 ± 6.3 mm (30 mg GR3027, *p* = 0.06). Sedation levels before and after GR3027 were very similar and not-statistically different; a significant ~ 3-mm increase in sedation was seen by placebo treatment.

### Exposures to GR3027 and allopregnanolone

The GR3027 plasma concentration after 3 mg oral GR3027 was 102 ± 14 nM just before the injection of allopregnanolone. The GR3027 concentration was then relatively stable during the period with high allopregnanolone exposure, with a 20% decrease during that hour. With 30 mg oral GR3027, the plasma concentration was 1294 ± 106 nM at the time for the allopregnanolone injection and with a similar kinetic profile as for the low dose (supplementary Fig. 2).

At the three study occasions, the serum allopregnanolone concentration curves were very similar (supplementary Fig. 2) with the highest serum level at the first study time point after the i.v. injection, 74–82 nM. The allopregnanolone concentrations thereafter quickly decreased and were almost back to baseline 3 h after the injection. There were no significant differences between the allopregnanolone exposures during the three study occasions (*p* = 0.16, comparisons of AUC_t_ allopregnanolone).

Subjects with a BMI of 25 or higher (*n* = 7) had a significantly higher allopregnanolone concentration at the first studied time point, 88 ± 2.8 nM, compared to those with BMI < 25 (*n* = 10), 66 ± 2.7 nM (*p* = 0.001, Mann-Whitney *U* test, mean BMI 24, range 20–28 kg/m^2^). However, there was no significant difference between the BMI groups when serum allopregnanolone over the whole study period was analyzed (*p* = 0.143, Mann-Whitney *U* test).

## Discussion

The present findings demonstrate that orally administered GR3027 is well tolerated in humans. AEs were generally mild and neither SAEs nor dose-limiting toxicity was observed up to the highest doses administered to healthy adult males in the single (200 mg) or multiple (100 mg every 12 h for 5 days) ascending dose protocols. At all doses studied, GR3027 exhibited linear PK, i.e., both *C*_max_ and systemic exposure assessed as AUC_0–∞_ (SAD) or AUC_τ_ (steady-state in MAD) were linearly related to oral dose. During steady-state dosing, accumulation ratios were dose dependent and ranged from 1.3 to 1.6.

Importantly, the results of the challenge study further indicate that GR3027 enters the brain and antagonizes the effects of allopregnanolone at the GABA-A receptor. By prespecified analysis, GR3027 at 30 mg, but not 3 mg, significantly inhibited the effects of allopregnanolone on SEV, whereas neither doses had a significant effect on sedation. However, based on post hoc analyses restricted to just those subjects who exhibited an allopregnanolone-induced change in SEV or self-rated sedation and to just the time interval during which these effects were observed, orally administered GR3027 produced apparent dose-dependent inhibition of the effects of allopregnanolone. SEV was significantly inhibited by 30 mg GR3027 with an intermediate, but non-significant, inhibition at 3 mg. GR3027 also significantly reversed allopregnanolone-induced sedation at both the 3 and 30 mg doses, again in apparent dose-dependent fashion.

The prespecified analyses in the present study were based on prior studies of allopregnanolone administration to healthy adult females (Bengtsson et al. 2015; Hedstrom et al. 2015; Timby et al. 2016). While the effects of the allopregnanolone challenge observed in the present study in healthy males, including the maximal change in both SEV and sedation, are generally similar to those previously described in healthy adult females (Bengtsson et al. 2015; Hedstrom et al. 2015; Moller et al. 2016; Timby et al. 2016), interesting differences were observed. For example, only 50–70% of the healthy adult male subjects exhibited sedation and/or SEV inhibition in the present study vs. over 90% of healthy adult females given the same milligram per kilogram dose of allopregnanolone (unpublished data). Moreover, among those males who did respond to allopregnanolone, the effect lasted for a shorter period of time, i.e., ~ 80 min for both SEV and sedation, vs. 120 to more than 180 min in females given similar doses (Hedstrom et al. 2015; Moller et al. 2016; Timby et al. 2016). Although decreased sensitivity to allopregnanolone-induced changes in SEV has been observed in overweight females (Hedstrom et al. 2015), response among males to allopregnanolone in the present study appeared unrelated to either BMI or serum allopregnanolone levels.

We are aware of one prior study (van Broekhoven et al. 2007) in which both men and women received i.v. allopregnanolone that suggested the presence of gender-related differences. At the highest dose (0.045 mg/kg), males exhibited a smaller SEV response than females while no gender difference in SEV response was reported at lower doses. By contrast, males exhibited more sedation than females in response to low doses of allopregnanolone, but also seemed to recover more quickly than females. In the von Broekhoven study (van Broekhoven et al. 2007), serum allopregnanolone concentrations increased significantly more in males vs. females. These findings in aggregate suggest that while the overall response to an allopregnanolone challenge is similar among healthy adult males and females, there are likely subtle gender-related differences in allopregnanolone uptake and/or disposition and/or GABA-A receptor sensitivity to allopregnanolone that account for the differences in the response to allopregnanolone exhibited by males vs. females (Backstrom et al. 2013; Hedstrom et al. 2015; Timby et al. 2016).

The favorable safety and pharmacokinetic findings as well as the results of the allopregnanolone challenge study suggest that GR3027 represents a promising new treatment for human disorders attributable to the CNS effects of neurosteroids. Hepatic encephalopathy (HE), a neurological disorder associated with cirrhosis and portal-systemic shunting in which elevated brain levels of allopregnanolone have been reported (Ahboucha et al. 2005; Ahboucha et al. 2006), is one example. The manifestations of HE comprise a wide spectrum that ranges from subtly impaired covert neuropsychiatric abnormalities detectable only by standardized testing to clinically overt abnormalities that include altered motor function, sleep disturbances with changes in the sleep-wake cycle, daytime drowsiness, slurred speech, inappropriate behaviour, impaired memory impairment, shortened attention span, altered mental status with disorientation, coma, and even death (Bajaj et al. 2011; Montagnese et al. 2015; Vilstrup et al. 2014). Indeed, GR3027 has shown promise in two different animal models of HE, including rats with portacaval shunts and rats with chronic hyperammonemia, both of which are associated with increased brain concentrations of neurosteroids (Ahboucha et al. 2008; Cauli et al. 2009). In both animal models, GR3027 restored motor coordination, spatial learning, and spatial memory and partially restored the abnormal circadian rhythm in portacaval shunt rats (Johansson et al. 2015).

The apparently greater effect of GR3027 on reversing allopregnanolone’s effect on sedation vs. SEV in the present study in adult males is an interesting finding reminiscent of prior findings in the portacaval shunt rat model of HE (Johansson et al. 2015), in which a lower subcutaneous dose of GR3027 restored motor coordination compared to the higher dose needed to restore learning in the radial maze. One potential explanation for this differing dose-dependency is that different areas of the brain with differing GABA-A receptor subtypes are responsible for different neurological functions, i.e., SEV is specifically controlled by GABA-A receptors in the superior colliculus and in cerebellum (Hikosaka and Wurtz 1983; Kojima et al. 2010), while spatial learning is mainly modulated in the hippocampus (Moser et al. 2017). It is also possible that uptake and/or disposal of allopregnanolone or GR3027 differs among differing brain areas (Bixo et al. 1997; Johansson et al. 2015; Wang et al. 1995).

The findings of the challenge study extend the earlier findings in animal models. While the CNS concentrations of allopregnanolone resulting from the i.v. challenge cannot be directly measured, currently available data suggest that they are pathophysiologically relevant to those in human disease states and that GR3027 antagonizes the effects of allopregnanolone at such concentrations. Serum allopregnanolone concentrations measured in adult males 10 min after injection averaged ~ 80 nM, considerably higher than concentrations of 6–20 nmol/kg in the brain of cirrhotic patients who died in hepatic coma (Ahboucha et al. 2005; Ahboucha et al. 2006). Moreover, animal studies indicate that the concentration vs. time profile for GR3027 (unpublished data) and allopregnanolone (Johansson et al. 2002) is very similar in brain and plasma. The results imply that GR3027 administered at doses as low as 30 mg, well within the range that is well tolerated in adult males, can reverse the effects of neurosteroids even at concentrations higher than those described in HE patients. Importantly, GR3027 by itself had no effect on either SEV or sedation. This is consistent with in vitro findings and its mechanism of action whereby it would be expected to selectively reverse only neurosteroid-mediated modulation of GABA-A receptors.

Several recent observations also implicate neurosteroid-induced allosteric activation of CNS GABA-A receptors in the excessive daytime sleepiness (EDS) associated with disorders such as idiopathic hypersomnia (Billiard and Sonka 2016). These include the identification of a population of GABAergic neurons in the ventral lateral hypothalamus that help mediate human wakefulness (Venner et al. 2016), the identification of a putative GABA-A agonist in the CSF of patients with primary hypersomnia (Rye et al. 2012), and the clinical observation that the GABA-A antagonist flumazenil can promote wakefulness in patients with EDS (Trotti et al. 2016). Indeed, the clinical manifestations of HE and hypersomnolence disorders exhibit similarities, and HE has been considered a sleepiness disorder (Montagnese et al. 2015).

In summary, these findings show that GR3027 administered orally to humans antagonizes the effects of the neurosteroid allopregnanolone at the GABA-A receptor in the brain at doses which are well tolerated and associated with linear pharmacokinetics. This suggests that GR3027 may also be able to normalize GABAergic neurotransmission and represents a promising new therapeutic for treatment of HE, disorders associated with hypersomnolence or other disorders attributable to neurosteroid-mediated allosteric over-activation of GABA-A receptors.

## Electronic supplementary material

ESM 1 (DOCX 201 kb)
