# Supplementary material for: GR3027 reversal of neurosteroid-induced, GABA-A receptor-mediated inhibition of human brain function: an allopregnanolone challenge study
Source: Psychopharmacology (Berl). 2018 Feb 28;235(5):1533–43. doi: 10.1007/s00213-018-4864-1 (PMC5919995; doi:10.1007/s00213-018-4864-1)
Supplement: Supplementary file 1 — (DOCX 201 kb) [file 213_2018_4864_MOESM1_ESM.docx]

Supplementary material Psychopharmacology

**GR3027 reversal of neurosteroid-induced, GABA-A receptor-mediated inhibition of human brain function: an allopregnanolone challenge study**

Maja Johansson^1,2^, Maria Månsson^1^, Lars-Eric Lins^1^, Bruce Scharschmidt^1^, Magnus Doverskog^1^, Torbjörn Bäckström^1,2
1^Umecrine Cognition AB, Karolinska Institutet Science Park, Fogdevreten 2, SE-171 65 Solna, Sweden; ^2^Department of Clinical Sciences, Obstetrics and Gynecology, Umeå University, SE-901 87 Umeå, Sweden.

**Corresponding author** Maja Johansson Email: Maja.Johansson@Umecrine.se


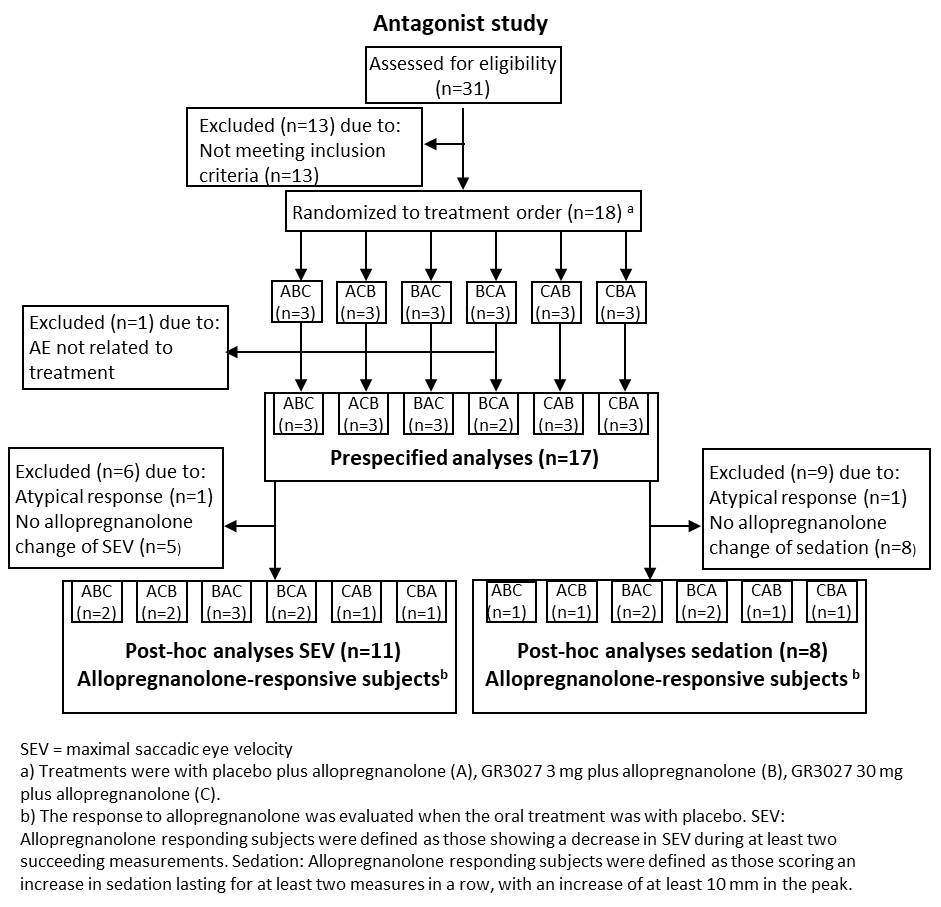


Supplement Fig. 1 Disposition of subjects in the antagonist study

Supplement **Fig. 2** Concentrations of GR3027 in plasma after oral GR3027 3 mg (n=18) and 30 mg (n=17), and concentrations of allopregnanolone in serum at the 3 study occasions of the antagonist study (n=17 with vehicle and with 30 mg GR3027, n=18 with 3 mg GR3027). Note that concentrations are presented as nmol/L

**Supplementary Table 1** Plasma GR3027 pharmacokinetic parameters in the SAD and MAD studies, data are presented as mean (SD)**.**

| **GR3027**  **treatment** | **C_Max_**  **(ng/mL)** | **T_Max_**  **(h)** | **AUC_0-∞_ (h*ng/mL)** | **AUCτ**  **(h*ng/mL)** | **T_1/2_**  **(h)** | **CL/F**  **(L/h)** | **R_A,AUC_** |
| --- | --- | --- | --- | --- | --- | --- | --- |
| Single dose:  1 mg  3 mg  10 mg  30 mg  100 mg  300 mg | 13 (3.2)  44 (8.6)  149 (18)  464 (58)  1525 (669)  2775 (491) | 1.2 (0.3)  1.3 (0.5)  0.9 (0.2)  1.3 (0.5)  1.8 (0.7)  2.2 (0.7) | 67 (13)  215 (16)  785 (178)  2565 (291)  11861 (3357)  25827 (7080) |  | 6.0 (1.8)  8.7 (3.2)  6.4 (1.3)  7.7 (1.7)  9.8 (2.2)  9.2 (2.2) | 15.4 (2.5)  14.0 (1.1)  13.3 (2.9)  11.8 (1.2)  9.0 (2.6)  8.3 (2.4) |  |
| Multiple dose  (first dose, day 1):  50 mg  BID 50 mg  BID 100 mg  Multiple dose  (steady-state, day 5):  50 mg  BID 50 mg  BID 100 mg | 772 (131)  911 (183)  1314 (388)  892 (188)  924 (256)  1440 (204) | 1.6 (0.4)  1.3 (0.4)  2.4 (1.8)  1.7 (0.4)  1.8 (1.1)  2.7 (0.8) | 4894 (1098)  4293 (989)  8201 (1357)  6162 (1279)  9167 (4486)  18768 (6174) | 4121 (824) ^a^  3486 (659) ^b^  6134 (928) ^b^  5174 (857) ^a^  5202 (1585) ^b^  9898 (1607) ^b^ | 10.2 (3.5)  5.1 (1.2)  6.1 (3.0)  9.7 (3.3)  12.0 (3.5)  12.0 (4.6) | 10.7 (2.9)  12.2 (2.8)  12.4 (2.0) | 1.3 ± 0.1  1.5 ± 0.3  1.6 ± 0.4 |

^a^ τ = 24 hours, ^b^ τ = 12 hours

C_Max_ (maximal concentration), T_Max_ (timepoint for C_max_), AUC_0-∞_ (area under the curve extrapolated to infinity), AUCτ (area under the curve during dosing interval ), T_1/2_ (terminal half-life), CL/F (total apparent body clearance following extravascular administration), R_A,AUC_ (accumulation ratio for AUCτ)
